# Dual Beam In Situ Radiation Studies of Nanocrystalline Cu

**DOI:** 10.3390/ma12172721

**Published:** 2019-08-25

**Authors:** Cuncai Fan, Zhongxia Shang, Tongjun Niu, Jin Li, Haiyan Wang, Xinghang Zhang

**Affiliations:** 1 School of Materials Engineering, Purdue University, West Lafayette, IN 47907, USA; 2 School of Electrical and Computer Engineering, Purdue University, West Lafayette, IN 47907, USA

**Keywords:** in situ TEM, dual-beam irradiation, nanocrystalline, grain coarsening, helium bubbles

## Abstract

Nanocrystalline metals have shown enhanced radiation tolerance as grain boundaries serve as effective defect sinks for removing radiation-induced defects. However, the thermal and radiation stability of nanograins are of concerns since radiation may induce grain boundary migration and grain coarsening in nanocrystalline metals when the grain size falls in the range of several to tens of nanometers. In addition, prior in situ radiation studies on nanocrystalline metals have focused primarily on single heavy ion beam radiations, with little consideration of the helium effect on damage evolution. In this work, we utilized in situ single-beam (1 MeV Kr^++^) and dual-beam (1 MeV Kr^++^ and 12 keV He^+^) irradiations to investigate the influence of helium on the radiation response and grain coarsening in nanocrystalline Cu at 300 °C. The grain size, orientation, and individual grain boundary character were quantitatively examined before and after irradiations. Statistic results suggest that helium bubbles at grain boundaries and grain interiors may retard the grain coarsening. These findings provide new perspective on the radiation response of nanocrystalline metals.

## 1. Introduction

Irradiation of metals and alloys produces supersaturated point defects (Frenkel pairs) and defect clusters [1,2], and thus leads to the degradation in their physical and mechanical properties [3,4]. One effective strategy to alleviate radiation damage is to use various types of interfaces [5], such as grain boundaries (GBs) [6,7], twin boundaries (TBs) [8,9], phase boundaries [10,11], and free surfaces [12,13]. These interfaces act as defect sinks and are expected to attract, absorb, and annihilate radiation-induced defects [14]. There are increasing evidences showing that nanostructured materials with high volume fraction of interfaces are more radiation-tolerant than conventional materials [15,16,17,18,19], in terms of lower defect density [20,21], less radiation-induced hardening [22,23], and stronger resistance against amorphization [24]. In spite of their enhanced radiation tolerance, nanostructured materials tend to become thermally unstable because of the extra energy stored at interfaces [25]. For instance, radiation-assisted GB and TB migration, accompanied by grain coarsening and detwinning, were reported in nanocrystalline (NC) [26,27] and nanotwinned (NT) metals [28,29,30,31], especially when the grain size or twin spacing reduces to several to tens of nanometers [29,32].

At the core of nuclear reactors, structural materials are exposed to intense fluxes of neutrons at elevated temperatures [33]. In order to investigate such radiation damage in a safe, economic, and efficient way, heavy ion irradiation technique was developed and has been widely adopted as a surrogate for emulating neutron irradiation damage in the past decades [34]. However, there are various challenges for the use of single heavy ion irradiation technique to emulate neutron-radiation-induced damage [35]. For instance, a single type of heavy ion irradiation study often lacks helium (He), inevitably arising from some nuclear reactions, like the D-T nuclear fusion reaction [36]. As an inert gas, He is hardly soluble in solids and plays an important role in microstructure evolution [37,38,39,40]. Under irradiation, He can easily combine with excess vacancies and precipitate as bubbles in matrix, dislocations, GBs, or heterointerfaces [41,42]. With increasing neutron fluxes and addition of He atoms, the bubbles may keep growing, leading to void swelling, hardening, and embrittlement [4,43,44,45]. Therefore, to better simulate the neutron radiation damage with heavy ion irradiation technique, it has been suggested that pre-injection or simultaneous implantation of He may be necessary while conducting regular heavy ion irradiation studies [46].

In this work, we utilize in situ transmission electron microscope (TEM) technique to directly compare the distinctions between single-beam heavy ion irradiation (1 MeV Kr^++^) and dual-beam irradiation (by 1 MeV Kr^++^ and 12 keV He^+^), and combine the recent advance in automated crystal orientation mapping capability in transmission electron microscope, to explore the He effect on ion irradiation-induced grain coarsening in NC Cu at 300 °C. The findings provide new insights for understanding the irradiation response of NC metals and their potential applications in advanced nuclear energy system.

## 2. Materials and Methods

**Figure 1:**
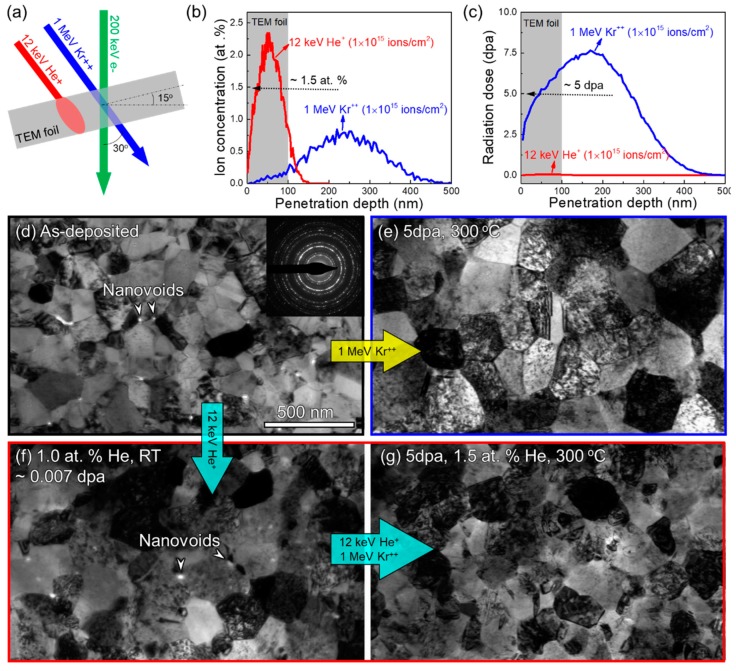
Singe- and dual-beam irradiations on nanocrystalline (NC) Cu at 300 °C. (**a**) Experimental set up of in situ heavy ion TEM irradiations; (**b**,**c**) SRIM calculations of depth profiles of ion concentration and corresponding radiation dose; (**d**) As-deposited NC Cu with some preexisting nanovoids at grain boundaries (GBs); (**e**) Single-beam (1 MeV Kr^++^) irradiation at 300 °C for 5 dpa; (**f**) He pre-injection at room temperature (RT); (**g**) Dual-beam (1 MeV Kr^++^ and 12 keV He^+^) irradiation at 300 °C to 5 dpa.

NC Cu (99.995 at.%) films (~2 μm) were deposited on the HF-etched Si (111) substrates at room temperature (RT) by direct current magnetron sputtering technique. Plan-view TEM specimens were prepared and subsequently irradiated using the Intermediate Voltage Electron Microscope (IVEM) Tandem Facility at the Argonne National Laboratory (Chicago, IL, USA), where an ion accelerator is attached to a Hitachi 900 NAR microscope (Hitachi, Tokyo, Japan) operated at 200 kV. The ion source included 1 MeV Kr^++^ and 12 keV He^+^ with the ion beam incidented at 30° from the electron beam and 15° from the foil normal, as schematically illustrated in Figure 1a. To explore the effect of He on radiation damage, two independent irradiation experiments were conducted using the single heavy ion beam of Kr^++^, and the dual beams of Kr^++^ plus He^+^. For single-beam irradiation, the specimen was irradiated by 1 MeV Kr^++^ at 300 °C at a dose rate of 6.25 × 10^11^ ions cm^−2^ s^−1^ up to a fluence of 1 × 10^15^ ions cm^−2^. For dual-beam irradiation, the specimen was first implanted at RT by 12 keV He^+^, with a dose rate of 1.25 × 10^12^ ions cm^−2^ s^−1^ and to a fluence of 6.7 × 10^14^ ions cm^−2^. The He-injected specimen was then irradiated simultaneously by 1 MeV Kr^++^ and 12 keV He^+^, and their dose rates were 6.25 × 10^11^ ions cm^−2^ s^−1^ and 2.08 × 10^10^ ions cm^−2^ s^−1^, respectively, up to the same fluence of 1 × 10^15^ ions cm^−2^.

Irradiation damage was calculated by the Stopping and Range of Ions in Matter (SRIM) with full damage cascades and the displacement energy of 30 eV for Cu [47]. The calculated depth profiles of ion concentration and radiation damage, in unit of displacements-per-atom (dpa), are given in Figure 1b,c. The TEM foil thickness is estimated to be ~100 nm, and the SRIM calculations reveal that most (~95%) of the Kr^++^ transmitted through the TEM foil and caused a high radiation dose of ~5 dpa, while most (~92%) of the He^+^ ions were injected into the foil with negligible damage, ~0.01 dpa. The average He concentration is ~1.5 at.%.

All the TEM specimens, as-deposited or irradiated, were characterized by a Thermo Fischer Scientific/FEI Talos 200X microscope equipped with a NanoMEGAS ASTAR precession electron diffraction system that allows for high-resolution crystal orientation mapping [48]. Multiple locations with the same area (1.92 × 1.92 μm^2^) were selected and scanned for each specimen by using ASTAR system. The spot size of electron beam for scanning is ~2 nm, and the scanning step size is ~5 nm.

## 3. Results

### 3.1. In Situ Study of Irradiation-Induced Microstructure Evolution

Figure 1d is a bright-field (BF) TEM micrograph of the as-deposited sample with a broad distribution of grain sizes, ranging from tens of nm to a few hundred nm. The inset selected area diffraction (SAD) pattern indicates the formation of polycrystalline metals, and the arrows denote some nanovoids formed along GBs. Figure 1e shows the microstructure after single-beam irradiation by 1 MeV Kr^++^ to 5 dpa at 300 °C. Compared with Figure 1d, grain sizes in Figure 1e have apparently increased and the preexisting nanovoids have disappeared. Figure 1f shows the microstructure after He-injection to a concentration of 1 at.% at RT and a low dose of only 0.007 dpa. Most of the preexisting nanovoids retained. After irradiations with dual beams of 1 MeV Kr^++^ and 12 keV He^+^ to 5 dpa at 300 °C, corresponding to a He concentration of 1.5 at.%, Figure 1g displays that most of the nanovoids have disappeared. In addition, the average grain size after dual-beam irradiation in Figure 1g seems to be between that of as-deposited specimen in Figure 1d and that of single-beam irradiated specimen in Figure 1e.

**Figure 2:**
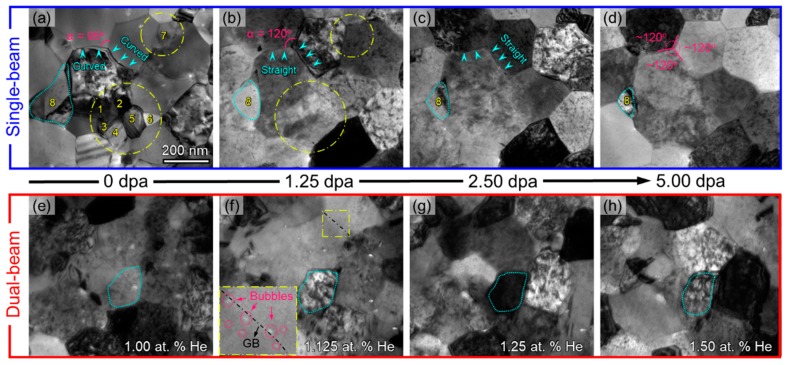
In situ TEM snapshot displaying microstructural evolution of NC Cu under single-beam (**a**–**d**) and dual-beam irradiation (**e**–**h**). GB migrations were frequently captured in single-beam irradiation and grain coarsening occurred at the expense of small grains, as evidenced by the shrinkage of several tiny grains marked by number 1–8 in (**a**–**d**). The arrows in (**a**) mark the curved GBs for a large grain that became straight with increasing dose in (**b**, **c**). In contrast, the grains under dual-beam irradiation only experienced slight rearrangement of their geometries, as shown by the dotted lines in (**e**–**h**).

Figure 2 compares the TEM snapshots of NC Cu subjected to single-beam and dual-beam irradiations to 5 dpa. Frequent GB migrations of small grains were captured in single-beam irradiation in Figure 2a–d, and the shrinkage rate of small grains tended to decrease with increasing grain size. For instance, 7 representative tiny grains that are <100 nm are denoted by 1–7 in Figure 2a, and they all shrank rapidly and disappeared when irradiated to 1.25 dpa, as shown in Figure 2b. In contrast, another large grain marked as 8 in Figure 2a–d remained its triangular shape and shrank gradually. Moreover, several other large grains barely shrank but evolved into polygons. Their initially curved GBs became straight, as marked by the arrows in Figure 2a–c. Meanwhile, the angles between adjacent grains evolved to an equilibrium angle of ~120°, as shown in Figure 2d. In comparison, no obvious GB migrations were observed in dual-beam irradiated Cu shown in Figure 2e–h. Some of the grains slightly rearranged their geometry as shown by a typical outlined grain in Figure 2e–h. He bubbles emerged at 1.25 dpa with a He concentration of ~1.125 at.%, as shown by the inset in Figure 2f.

### 3.2. Post-irradiation Analyses

**Figure 3:**
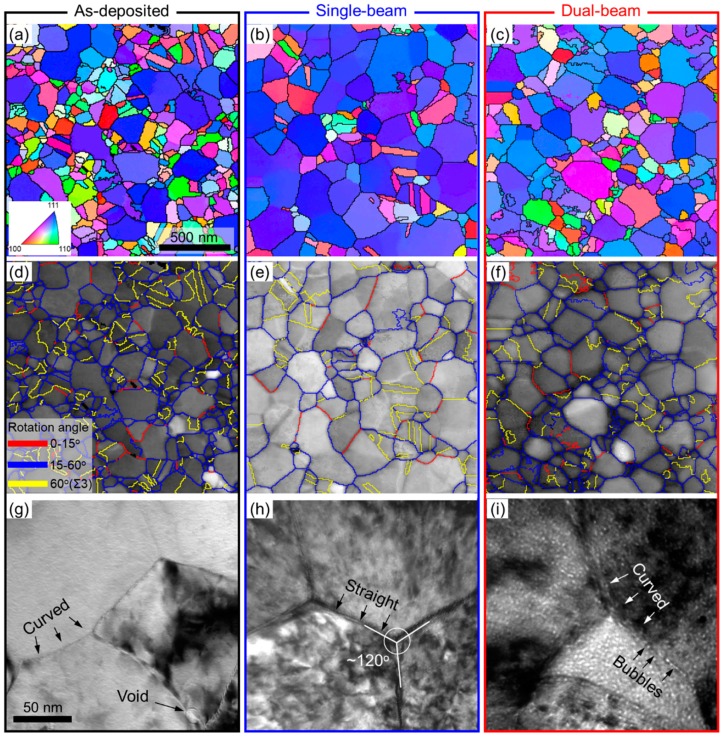
Microstructural characterization of as-deposited (**a**,**d**,**g**), single-beam irradiated (**b**,**e**,**h**), and dual-beam irradiated samples (**c**,**f**,**i**). (**a**–**c**) Crystal orientation maps obtained from ASTAR system. (**d**–**f**) Grain boundary maps superimposed upon image quality maps. (**g**–**i**) TEM micrographs showing enlarged views of representative GBs.

To better characterize the evolution of GBs, an ASTAR automated crystal orientation mapping system was used to analyze the as-deposited, single-beam irradiated, and dual-beam irradiated Cu samples. Comparison of the orientation maps in Figure 3a–c clearly demonstrates prominent grain growth in irradiated specimens. In addition, the grain boundary maps in Figure 3d,f reveal a large fraction of Σ3 coherent TBs (yellow lines) in all samples. The enlarged view in Figure 3g reveals that the as-deposited NC Cu is characterized by irregular and curved GBs. In comparison, the single-beam irradiated NC Cu contains a significant number of straight boundaries often forming angles of 120° at triple junctions, as shown in Figure 3h. The dual-beam irradiated NC Cu, on the other hand, maintains curved GBs that are decorated with abundant He bubbles as shown in Figure 3i.

**Figure 4:**
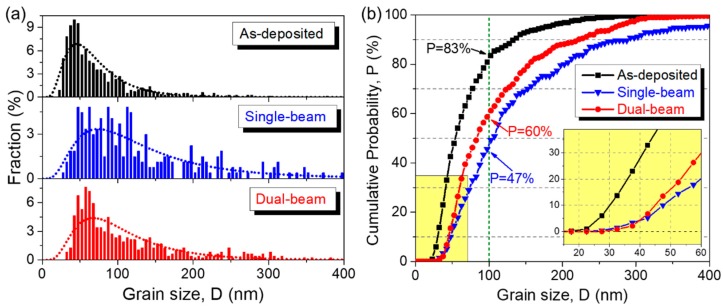
(**a**) Grain size (equivalent diameter) histograms for as-deposited (black), single-beam irradiated (blue), and dual-beam irradiated (red) sample. (**b**) Cumulative probability versus grain size.

The statistics of grain size evolutions were derived from a study of 1646 grains for the as-deposited sample, 552 and 696 grains for the single-beam and dual-beam irradiated samples, respectively. The grain size is quantified by equivalent diameter D that equals to 2√(A/π), where A refers to the individual grain area. The grain size histograms for the three samples are shown in Figure 4a, and their corresponding cumulative probabilities P are plotted in Figure 4b as a function of D. Note that the probability curves shift rightward after irradiations due to grain growth, and the red curve for dual-beam irradiated sample in Figure 4b is between that of as-deposited (black) and single-beam irradiated sample (blue). The fraction of smaller grains that are less than 100 nm drops from 83% to 60 and 47% after dual-beam and single-beam irradiation, respectively. The corresponding median grain size $D_{0.5}$ (P=0.5) increases from 56 ± 4 nm to 83 ± 2 nm after dual-beam irradiation, and to 103 ± 5 nm after single-beam irradiation. In particular, the lower right inset in Figure 4b reveals that the fraction of grains smaller than 40 nm is around 30% in the as-deposited sample, and it decreases drastically after either dual-beam or single-beam irradiation.

**Figure 5:**
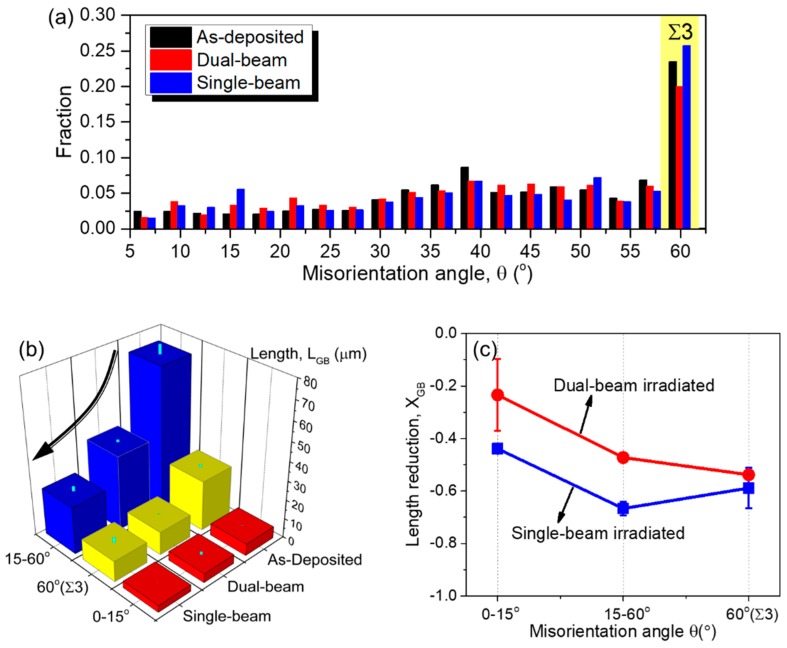
(**a**) Misorientation angle (θ) histograms for as-deposited (black), single-beam irradiated (blue), and dual-beam irradiated (red) samples. (**b**) Boundary length for low-angle (red bars, θ = 0–15°) and high-angle (blue bars, θ = 15–60°) GBs, as well as Σ3 twin boundaries (TBs) (yellow bars, θ = 60°). (**c**) GB length reduction for dual-beam (red) and single-beam (blue) irradiated samples, relative to the as-deposited sample.

The misorientation angle (θ) distributions for the three specimens are compared in Figure 5a. The GBs have been divided into three major categories according to their misorientation angle: low-angle GBs (θ < 15°), high-angle GBs (15° < θ < 60°), and special Σ3 coherent TBs (θ = 60°). TBs account for one-quarter of all boundaries for the three specimens. The statistic results in Figure 5b,c show that all GBs decreased in length after irradiations, and it was found that the high-angle GBs (15° < θ) in single-beam irradiated sample reduced the most. The detailed statistics on evolutions of grain size and GB misorientation angles are summarized in Table 1.

## 4. Discussion

Grain coarsening arises from GB migration. The migration velocity v of an isolated boundary in one dimension can be described by [49]:

(1) $$v=-M\frac{\partial \mu }{\partial x}$$

where M is the GB mobility and increases with increasing temperature, and ∂μ/∂x is the driving force and increases with decreasing grain size due to the boundary curvature effect. There are increasing experimental evidences that show GB migration velocity is accelerated considerably under irradiation [27,32,50,51], and the irradiation-enhanced grain coarsening occurs even at room temperature when thermal activation makes little contribution [26]. To describe the radiation effects on grain coarsening, a thermal spike (damage cascade) model was proposed [26,49], according to which the radiation-assisted GB migration occurs within thermal spikes through atomic jumps that are biased by local GB curvature. Moreover, the radiation effects on GB structure and its migration were also well studied by atomistic simulations [6,50,52,53,54,55]. It was found that the free volume in GBs can accommodate extra interstitials [6,52], which makes interstitial-loaded GBs so unstable that they frequently migrate to annihilate vacancy clusters nearby [53,54]. In addition, for small grains with dimensions comparable to the thermal spike volume, their boundary area may overlap with thermal spikes, so small grains may undergo drastic grain growth through disorder-driven mechanism [32,55]. These prior studies suggest that irradiation-induced grain growth is often determined by two major factors: the GB curvature and the interaction between damage cascades and GBs. In the current study, we found that the radiation-assisted grain coarsening can also be influenced by the He bubbles, and the underlying mechanism will be discussed later in detail.

The interaction between damage cascades and GBs can be simply divided into two scenarios that are sink-dominated or recombination-dominated. For the former scenario, the majority of radiation-induced defects, including equal numbers of vacancies and interstitials, are trapped and annihilated by defect sinks; whereas for the latter case, defects are mostly eliminated through vacancy-interstitial recombination. It was proposed that a dimensionless parameter E that considers the defect recombination and fluxes into defect sinks can be used to evaluate which mechanism is dominating [26]. In the single-beam irradiation, the parameter $E_{1}$ can be written as:

(2) $$E_{1}=\frac{\left(k_{GB}^{2}D_{i}\right)\left(k_{GB}^{2}D_{v}\right)}{4K_{0}K_{iv}}$$

where $D_{i}$ and $D_{v}$ are the diffusion coefficients for interstitials and vacancies, respectively. $k_{GB}^{2}$ is the GB sink strength, estimated as [56]:

(3) $$k_{GB}^{2}=\frac{60}{D^{2}}$$

where D is the grain size. $K_{0}$ is the displacement rate (~0.003 dpa/s in current study), and $K_{iv}$ is the constant for interstitial-vacancy recombination rate and is given by:

(4) $$K_{iv}=\frac{4\pi \left(D_{i}+D_{v}\right)}{\Omega }=K_{iv_{0}}\left(D_{i}+D_{v}\right)$$

where Ω is the atomic volume, and $K_{iv_{0}}$, in cm^−2^, is a material constant, ~6.98 × 10^16^ cm^−2^ for Cu [26]. Substituting Equations (3) and (4) into Equation (2) yields:

(5) $$E_{1}=\frac{D^{4}K_{0}K_{iv_{0}}}{900}\left(\frac{1}{D_{i}}+\frac{1}{D_{v}}\right)$$

As $D_{i}\gg D_{v}$, for irradiation of Cu at 300 °C, Equation (5) is simplified to:

(6) $$E_{1}=\frac{D^{4}K_{0}K_{iv_{0}}}{900D_{v}}$$

Under dual-beam irradiation, He bubbles act as extra defect sinks that compete with GBs in absorbing point defects. The effective parameter $E_{2}$ in the presence of He bubbles is thus modified as:

(7) $$E_{2}=\frac{\left(k_{GB}^{2}D_{i}\right)\left(k_{GB}^{2}D_{v}\right)}{4K_{0}K_{iv}+\left(k_{B}^{2}D_{i}\right)\left(k_{B}^{2}D_{v}\right)}$$

where $k_{B}^{2}$ is the sink strength for bubbles and is given by [57]:

(8) $$k_{B}^{2}=\frac{4\pi \rho R^{2}}{a}$$

where ρ is the bubble density, R is the bubble radius, and a is the lattice parameter (~0.3615 nm for Cu). Combining Equations (3)–(5) and (8), Equation (7) can be simplified into:

(9) $$E_{2}=\frac{900D_{v}a^{2}}{a^{2}K_{0}K_{iv_{0}}+4\pi R^{4}\rho^{2}D_{v}D^{4}}$$

**Figure 6:**
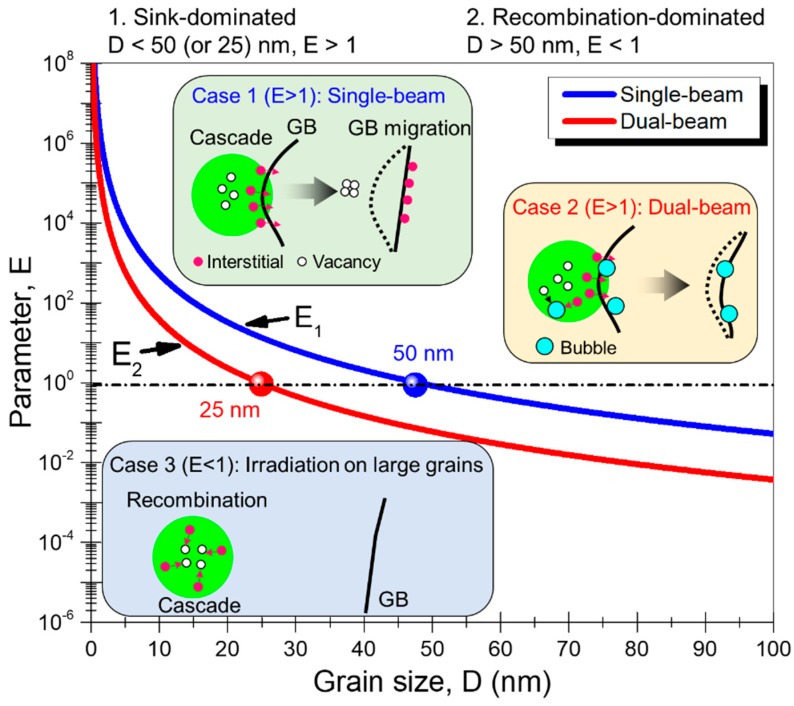
E parameter (logarithmic scale) versus grain size for single-beam (blue curve) and dual-beam (red curve) irradiated Cu at 300 °C. Case 1: Single-beam irradiation on small grains. Case 2: Dual-beam irradiation on small grains. Case 3: Irradiation of large grains. See the text for more details.

Post-irradiation TEM study in Figure 3i shows that the bubble radius R is ~1 nm, and the bubble density is around 0.0005 nm^−3^. The vacancy diffusivity $D_{v}$ is estimated by [57]:

(10) $$D_{v}=a^{2}\upsilon \exp\left(-\frac{E_{m}^{v}}{kT}\right)$$

where υ is the Debye frequency (~10^13^ s^−1^), $E_{m}^{v}$ is the vacancy activation migration energy (0.8 eV for Cu) [57], k is the Boltzmann constant, and T is the temperature (300 °C in current study). Substituting all the parameters into in Equations (6) and (9) and plotting the values of $E_{1}$ and $E_{2}$ as a function of grain size D result in Figure 6.

The physical meaning of E in our calculations refers to the magnitude of radiation-induced defects that can be trapped by preexisting GBs relative to the defects removed through recombination or He bubbles. According to previous studies, it is plausible to assume that only when sufficient defects diffuse into GBs, GBs can experience structure change and instability, followed by GB migration and grain coarsening [53]. In principle, a value of E≫1 indicates the radiation–GB interaction is sink-dominated, whereas E≪1 indicates the interaction is recombination-dominated. The curves obtained in Figure 6 suggest the interaction transits from sink-dominated regime to recombination-dominated regime with increasing grain size D. There is a critical grain size, below which the interaction is sink-dominated and the radiation-assisted grain coarsening is most likely to occur. Note that the red curve of dual-beam irradiation is below the blue curve, and the critical grain size shifts from 25 nm to 50 nm, when He bubbles are present. The plot also suggests that grain size D should be kept slightly larger than the transition value, so that the nanograins can effectively enhance radiation tolerance while retaining their structural stability.

It is worth pointing out that the two curves in Figure 6 show little difference when the grain size D is less than 5 nm. Our in situ observations in Figure 2 and post-irradiation statistics in Figure 4 also indicate that nanograins rapidly disappeared under single-beam or dual-beam irradiation. The damage cascade size $D^{*}$ of 1 MeV Kr^++^ irradiation on Cu is estimated to be 7 nm (see Appendix A). Therefore, the direct overlap of damage cascade with nanograins of similar dimension may lead to drastic grain coarsening at the expense of fine grains through disorder-driven mechanism [32,55].

Next, we compare the radiation-assisted GB migration and grain coarsening under single-beam and dual-beam irradiations. For simplicity, we divide the results into three cases according to the grain size D, as schematically illustrated in Figure 6.

Case 1: Radiation-assisted grain coarsening for small grains under single-beam irradiation. This case applies to sink-dominated interaction (*E* > 1), and damage cascade can entirely or partially overlap with small grains. Sufficient point defects can diffuse into GBs, leading to GB structure change and migration through disorder-driven [32,55] or thermal spike mechanism [26,49].

Case 2: Radiation-assisted grain coarsening for small grains under dual-beam irradiation. In this case, the radiation-GB interaction is still sink-dominated (*E* > 1). However, due to the formation of high-density He bubbles at GBs or grain interiors, the fraction of defects contributing to GB structure change and migration is reduced. Compared with single-beam irradiation, the radiation-assisted GB migration in dual-beam irradiated specimen is inhibited for two reasons: first, the bubbles at grain interiors can capture more point defects; second, the bubbles at GBs can exert pinning effect on GBs.

Case 3: Irradiation on large grains with recombination-dominated interactions (*E* < 1). In this case, most of the radiation-induced defects are removed through vacancy-interstitial recombination, so they make little contribution to GB migrations. For single-beam irradiation, damage cascade may occasionally occur near GBs. However, as the grain sizes are rather large, the local boundary curvature change due to cascade may be too small to drive prominent GB migrations [54]. The GB migrations may stop when GBs become straight, as shown in Figure 2. Meanwhile, the triple junctions can reach a stable state with three equal angles of ~120°, as shown in Figure 3h. For dual-beam irradiation, the local GBs can hardly move because of the pinning effect arising from He bubbles. As a result, GBs remain curved after irradiation, as shown in Figure 3i.

Finally, it should be emphasized that, in addition to grain size, radiation-GB interaction and GB migration may also be influenced by GB inherent structures [58]. For instance, compared with regular high-angle GBs, coherent TBs (CTBs) exhibit remarkable thermal stability after annealing to 800 °C [59], and they can retain their structural integrity during heavy irradiation [29,60]. Our post-irradiation analyses in Figure 3 also reveal a large fraction of surviving Σ3 CTBs in single-beam and dual-beam irradiated samples. A previous study on He ion irradiation response by Demkowicz et al. [61] suggested that TBs are not effective defect sinks, based on the observation that there were no defect denuded zones near TBs in He ion irradiated NT Cu. A thorough analysis may be beneficial to compare the density and dimension of He bubbles in bulk Cu irradiated to the same dose. Interestingly the atomistic simulations by Demkowicz et al. revealed that Σ3 CTBs can promote Frenkel pair recombination and decrease point defect production rate [61]. There are numerous prior studies that show TBs are effective defect sinks [8,31,62,63]. For instance, in Kr ion irradiated NT Ag, the density of stacking fault tetrahedrons is less in NT Ag with smaller twin spacing [62]. In other words, a clear size effect exists in irradiated NT metals. Recently, it was reported that less He bubbles are produced in NT Cu with nanovoids than annealed coarse-grained Cu, when subjected to identical He ion irradiation conditions at RT [63]. Similar phenomenon was also reported in the Fe ion radiation studies on NT austenitic stainless steel [8]. Meanwhile, there are increasing in situ radiation studies that show TBs engage, interact, and eliminate radiation-induced defect clusters [64,65]. For instance, an in situ radiation study on NT Ag showed that there is indeed a defect denuded zone near TBs based on the statistics of time accumulated defect cluster density [65]. These observations and current study may offer a new strategy for improving radiation tolerance while maintaining microstructural stability through the coupling of TB architectures with other defects sinks [60,66].

## 5. Conclusions

Nanocrystalline Cu films were irradiated with single-ion beam (1 MeV Kr^++^) and dual-ion beams (1 MeV Kr^++^ and 12 keV He^+^). Substantial GB migration and grain coarsening were captured in irradiated samples. The irradiation-induced GB migration is attributed to the interaction between damage cascade and preexisting GBs. With increasing grain size, the interaction transits from sink-dominated to recombination-dominated regime, and the radiation-assisted grain coarsening occurs in sink-dominated region at the expense of small grains. In situ radiation experiments also show that grain coarsening in dual-beam irradiated sample was retarded when He bubbles were introduced at GBs and grain interiors.

## Figures and Tables

**Table 1:** Summary of grain size and GB characters for as-deposited, dual-beam irradiated, and single-beam irradiated NC Cu, collected from a large area (11.06 μm^2^) at three different locations. $D_{0.5}$: the median grain size when *P* = 0.5.

Sample	Number of Grains	Grain Size D_0.5_ (nm)	$\mathrm{Grain}\;\mathrm{Boundary}\;\mathrm{Length},\;L_{GB}\;(\mu m)$		
			0–15°	15–60°	60° (Σ3)
As-deposited	1646	56 ± 4	6.2 ± 0.1	73.8 ± 4.4	26.2 ± 0.7
Dual-beam	696	83 ± 2	4.7 ± 0.2	38.9 ± 0.7	12.1 ± 0.1
Single-beam	552	103 ± 5	3.5 ± 0.1	24.6 ± 1.9	10.8 ± 2.0

## Appendix A. Estimation of radiation cascade size $D^{*}$

The radiation cascade size, $D^{*}$, is determined by incident particle energy, and the average cascade volume V is given by [57]:

(A1) $$V=\frac{4}{3}\pi {\left(\frac{1}{2}D^{*}\right)}^{3}=\frac{E_{D}}{NU_{a}}$$

where N is the atom density, ~8.5 × 10^22^ atoms/cm^3^ for Cu, and $U_{a}$ is the energy per atom that can be estimated from the melting temperature of the target, ~0.3 eV for Cu. $E_{D}$ in Equation (A1) refers to the damage energy stored in a cascade, given by [67]:

(A2) $$E_{D}=\frac{E_{T}}{1+fg\left(\varepsilon \right)}$$

and the inelastic energy loss is calculated using a numerical approximation to the universal function g(ε):

(A3) $$g\left(\varepsilon \right)=3.4008\varepsilon^{\frac{1}{6}}+0.40244\varepsilon^{\frac{3}{4}}+\varepsilon$$

(A4) $$f=0.1337\;Z_{1}^{\frac{1}{6}}{\left(\frac{Z_{1}}{A_{1}}\right)}^{\frac{1}{2}}$$

The ε in Equation (A3) refers to the reduced energy described as:

(A5) $$\varepsilon =\frac{A_{2}E_{T}}{A_{1}+A_{2}}\frac{A}{Z_{1}Z_{2}e^{2}}$$

(A6) $$A=A_{0}{\left(\frac{9\pi^{2}}{128}\right)}^{\frac{1}{3}}\left(Z_{1}^{\frac{2}{3}}+Z_{2}^{\frac{2}{3}}\right)$$

where $A_{0}$ is the Bohr radius (~0.053 nm), e is the electronic charge, $Z_{1}$ and $Z_{2}$ are the atomic numbers of the projectile and target, and $A_{1}$ and $A_{2}$ are the mass numbers of the atoms.

The term $E_{T}$ in Equation (A2) is the transferred energy to primary knock-on atom (PKA). For the incident particles of 1 MeV Kr^++^, classified as heavy slow ions, the inverse square potential is proper for calculating the average transferred energy, $E_{T}$, to primary knock-on atom (PKA) in the Cu lattice [57]. The description of $E_{T}$ is given by:

(A7) $$E_{T}=\sqrt{\gamma E_{i}T_{min}}$$

where $E_{i}$ is the incident energy (1 MeV), $T_{min}$ is the minimum transferred energy that is equal to Cu displacement energy $E_{d}$, ~30 eV, and γ is a mass ratio defined by atomic masses of incident particle m and lattice atom M in the form of:

(A8) $$\gamma =\frac{4mM}{{\left(m+M\right)}^{2}}$$

where m is 83.80 u for Kr, and M is 63.55 u for Cu.

Combining the Equations (A2)–(A8) yields $E_{D}=41.13$ keV, substituting which into Equation (A1) gives the average thermal spike volume V=162 nm^3^ and thermal spike size $D^{*}=7$ nm.
